# Differentiation of the High Night Temperature Response in Leaf Segments of Rice Cultivars with Contrasting Tolerance

**DOI:** 10.3390/ijms221910451

**Published:** 2021-09-28

**Authors:** Stephanie Schaarschmidt, Ulrike Glaubitz, Alexander Erban, Joachim Kopka, Ellen Zuther

**Affiliations:** Max-Planck-Institute of Molecular Plant Physiology, Am Mühlenberg 1, 14476 Potsdam, Germany; schaarschmidt@mpimp-golm.mpg.de (S.S.); glaubitz@mpimp-golm.mpg.de (U.G.); Erban@mpimp-golm.mpg.de (A.E.); kopka@mpimp-golm.mpg.de (J.K.)

**Keywords:** high night temperature, rice cultivars, leaf segments, sheath, metabolomics, RNA-Seq

## Abstract

High night temperatures (HNT) affect rice yield in the field and induce chlorosis symptoms in leaves in controlled chamber experiments. However, little is known about molecular changes in leaf segments under these conditions. Transcript and metabolite profiling were performed for leaf segments of six rice cultivars with different HNT sensitivity. The metabolite profile of the sheath revealed a lower metabolite abundance compared to segments of the leaf blade. Furthermore, pre-adaptation to stress under control conditions was detected in the sheath, whereas this segment was only slightly affected by HNT. No unique significant transcriptomic changes were observed in the leaf base, including the basal growth zone at HNT conditions. Instead, selected metabolites showed correlations with HNT sensitivity in the base. The middle part and the tip were most highly affected by HNT in sensitive cultivars on the transcriptomic level with higher expression of jasmonic acid signaling related genes, genes encoding enzymes involved in flavonoid metabolism and a gene encoding galactinol synthase. In addition, gene expression of expansins known to improve stress tolerance increased in tolerant and sensitive cultivars. The investigation of the different leaf segments indicated highly segment specific responses to HNT. Molecular key players for HNT sensitivity were identified.

## 1. Introduction

Rice (*Oryza sativa* L.) is a staple food for more than half of the world’s population and plays an important role in ensuring future food security, especially in Asia [1], Africa and the American continent [2]. Global climate change is a severe threat to global food production by the amplification of environmental stressors such as rising temperatures that negatively affect the yield of all major crops, including rice [3]. In recent decades, the global mean surface temperature has risen by an average of 0.85 °C and current models predict an increase of up to 3.7 °C by 2100 [3,4]. This temperature increase develops asymmetrically with a faster rise in the global night-time minimum compared to global day-time maximum temperature, leading to “high night temperature” (HNT) conditions [5,6,7]. Several studies showed that HNT negatively effects rice yield (e.g., [8,9,10]) and recent reviews extensively covered different phenological and physiological aspects of HNT stress in rice and wheat [11,12].

The wide natural variation of rice causes diverse responses to HNT stress for different cultivars in controlled [13,14,15,16,17] and field environments [10,18,19,20]. The classification of HNT sensitivity for individual cultivars in these studies was based on physiological parameters such as grain yield [10,19,20], head rice yield [13,14,18], spikelet fertility [16,17] or chlorosis [15]. Advantageously, chlorosis scoring allows a classification of HNT sensitivity already in the early vegetative stage and was used previously to cluster twelve rice cultivars into HNT-sensitive (high chlorosis damage), HNT-intermediate and HNT-tolerant (no chlorosis damage) groups [15]. Follow-up studies investigated metabolic and transcriptomic changes in these rice cultivars under HNT stress and differentiated molecular processes between members of the HNT sensitivity groups [21,22]. Despite the growing and recently reviewed knowledge of natural variation in HNT stress at the molecular level [23], little is known about the detailed HNT response of leaf segments and the development of leaf senescence under HNT stress, particularly in sensitive cultivars. Leaf senescence is a regulated process in plants which leads to the death of cells, tissues or the whole organ. A detailed understanding of this process is relevant to improve crop plant stress resilience and yield [24].

In this study, we selected six rice cultivars with different tolerances to HNT stress that were previously investigated on physiological as well as on metabolic and transcriptomic levels [15,21,22]. While sensitive cultivars showed clear stress symptoms, developing chlorosis and necrosis, almost no leaf phenotype different from the control was observed for the tolerant cultivars. For a better understanding of these molecular processes, leaf samples were collected and divided into leaf sheath and three segments of the leaf blade: base, middle and tip. Metabolite and transcript profiling were performed for each segment and possible molecular key players in HNT sensitivity were identified.

## 2. Results

Previously, different grades of chlorosis damage were reported for a selection of 12 rice cultivars at HNT stress conditions and used for an HNT sensitivity classification [15]. Chlorosis spots became visible around five cm from the tip and developed further into chlorosis and necrosis of the whole tip in sensitive cultivars. Metabolic profiles of whole leaves were clearly distinct between cultivars with different HNT sensitivity levels [15,22], but nothing is known about the detailed HNT response of different leaf segments. In this study, six rice cultivars from two subspecies and with contrasting sensitivity to HNT were selected for an independent investigation of the HNT response in different leaf segments. Leaf segment samples (sheath, base, middle, tip) were collected from each cultivar exposed to HNT or control conditions and used for metabolite profiling by gas chromatography mass spectrometry (GC-MS) as well as transcript profiling by RNA sequencing (RNA-Seq).

### 2.1. Sensitive, Intermediate and Tolerant Cultivars Showed Different Phenotypes under HNT

**Figure 1:**
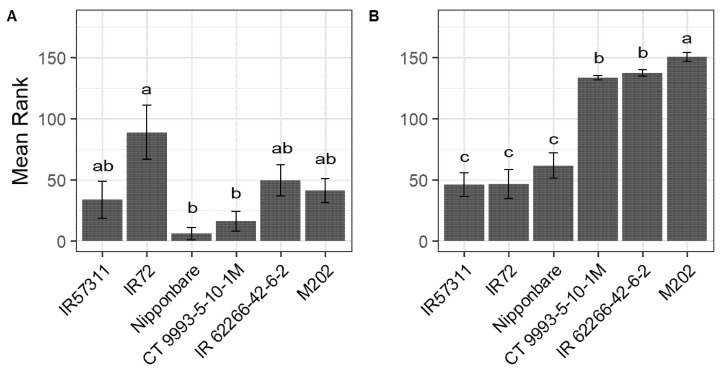
Mean rank of chlorosis scores of six *Oryza sativa* cultivars for control (**A**) and HNT (**B**) conditions. Cultivars are sorted based on chlorosis ranking under HNT. Larger values indicate high damage while smaller values represent low damage. Chlorosis mean ranks are based on visual scoring in percentage.

Six cultivars were classified as sensitive (IR62266-42-6-2, M202), intermediate (IR57311, CT9993-5-10-1M) and tolerant (IR72, Nipponbare) in previous studies [15,21,22]. For this study, chlorosis and necrosis levels as well as tiller number and leaf length were recorded in a new, independent experiment (Figure 1). The cultivars M202, IR62266-42-6-2 (both sensitive) and CT9993-5-10-1M (intermediate) had a significantly higher chlorosis level under HNT compared to IR57311 (intermediate), Nipponbare and IR72 (both tolerant) (Figure 1B), while for control conditions, less pronounced effects were observed (Figure 1A). The cultivar IR62266-42-6-2 (sensitive) showed the highest necrosis level under HNT, while the cultivars IR57311, CT9993-5-10-1M (both intermediate) and M202 (sensitive) were only intermediately damaged (Figure S1A). One intermediate (CT9993-5-10-1M) and one sensitive cultivar (M202) had a lower average change in leaf length compared to the two tolerant cultivars at HNT conditions (Figure S1B). Furthermore, in both intermediate and one sensitive cultivar (M202), a lower change in tiller number (Figure S1C) was detected under these conditions.

### 2.2. Metabolite Profile of the Sheath Differed from That of Leaf Blade Segments under Control Conditions

Little is known about the metabolite distribution in different rice leaf segments. Therefore, we compared log_2_-transformed metabolite levels of four leaf segments of six cultivars under control conditions by hierarchical clustering (Figure S2). All sheath, base, middle and tip leaf samples clustered together within the respective leaf segments, and only the IR57311 leaf middle sample clustered with the leaf base cluster. In general, the cultivars of the same subspecies *indica* or *japonica* grouped together within each leaf segment cluster.

Metabolites were sorted into six clusters (C1–C6) by hierarchical clustering. Cluster C1 and C2 showed similar tendencies with a high level of metabolites over all segments for all cultivars, with cluster C2 showing an overall lower level compared to C1. In cluster C2, especially the sugar raffinose, the unknown analyte A311002 as well as the sugar conjugate galactinol had a higher level in leaf sheaths compared to the remaining segment samples. The largest cluster C3 clearly differentiated metabolite levels in sheaths from the three other leaf segments with a remarkable lower abundance of all respective metabolites in leaf sheaths. This cluster included sugars (e.g., sucrose, maltose, arabinose, xylose), amino acids (e.g., glutamine, glutamic acid, aspartic acid, alanine, glycine), several organic acids (e.g., fumaric acid, shikimic acid, isocitric acid, glyceric acid) and unknown analytes. Cluster C4 contained only two metabolites, phenylalanine and gluconic acid, with low metabolite levels over all samples. Metabolites in cluster C5 depicted an increasing gradient from the lowest values in leaf sheaths to the highest values in leaf tip segments. This cluster included four unknown analytes, two polyols (erythritol, mannitol), one N-compound (putrescine) as well as the amino acid threonine and the sugar ribulose. The last cluster C6, including mainly amino acids, was characterized by very low metabolite abundance differences, except for galactaric acid, with higher levels in *indica* compared to *japonica* cultivars.

### 2.3. Leaf Segments Have Distinct Metabolite Profiles under HNT Stress

**Figure 2:**
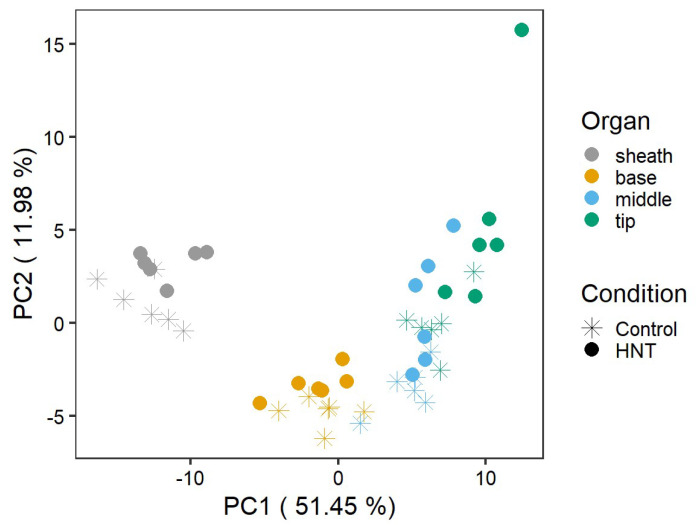
Principle component analysis of metabolite abundances normalized to internal standard and dry weight for leaf sheath, base, middle and tip segments of six cultivars. Shown are the scores of log_2_ median transformed data as mean of five replicates.

A global analysis of the metabolite data by PCA revealed a clear separation of the leaf segments (PC1) as well as the condition per segment (PC2), explaining either 51.45% or 11.98% of the variance (Figure 2). The leaf blade segments base, middle and tip clustered more closely together and separated from the sheath.

To investigate metabolic differences in more detail, the significance of changes under HNT compared to control conditions was determined for each metabolite and leaf segment. For the analyses, metabolites with missing values and unknown analytes were removed. Additionally, only metabolites with a significant change in at least two out of six cultivars were displayed, resulting in 62 metabolites in sheaths, 66 in the leaf base, 76 in the middle of the leaf and 81 in leaf tips. For a complete overview of all metabolic changes in all leaf segments and cultivars, see Figure S3.

**Figure 3:**
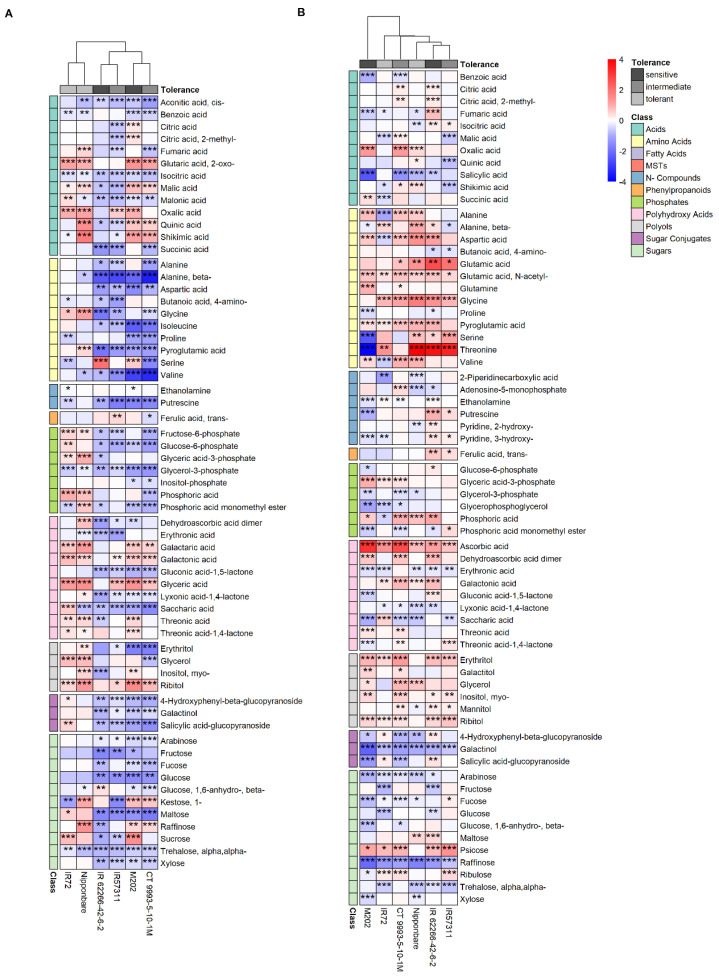
Changes in metabolite levels in leaf sheath (**A**) and base (**B**) segments represented as log_2_ fold changes. Red color indicates a higher log_2_ fold change, blue color a lower log_2_ fold change compared to the control. Asterisks display significance levels of *p* values: <0.001—***, ≥0.001 and ≤0.01—**, ≥0.01 and ≤0.05—*. Tolerance and metabolite classes are color-coded. For a better overview, only known metabolites with significant changes in at least two cultivars are shown.

In leaf sheaths (Figure 3A), most of the metabolites had a lower abundance under HNT compared to control conditions, except for some metabolites with common increased abundance in tolerant cultivars, mainly organic acids and sugar phosphates. Tolerant cultivars clustered together, while the sensitive and intermediate genotypes were grouped by their subspecies. When investigating tolerance differences between the cultivars, the metabolite classes amino acids (alanine, aspartic acid, isoleucine, pyroglutamic acid), phosphates (fructose-6-phosphate, glucose-6-phosphate, glycerol-3-phosphate, phosphoric acid) and sugars/sugar conjugates (4-hydroxyphenyl-beta-glucopyranoside, galactinol, salicylic acid-glucopyranoside, arabinose, fructose, glucose, maltose, xylose) had significantly lower levels in sensitive and intermediate cultivars compared to the tolerant ones when compared to their respective controls.

For samples of the leaf base, a different cultivar clustering compared to leaf sheaths was observed with no distinct grouping of tolerance classes or subspecies (Figure 3B). However, the polyols erythritol and ribitol, the polyhydroxy acid ascorbic acid, the sugar psicose and the amino acids N-acetyl-glutamic acid, glycine as well as the amino acid derivate pyroglutamic acid accumulated significantly higher in five or all cultivars under HNT compared to control conditions. Only the sugars arabinose and raffinose as well as the sugar conjugate galactinol were significantly less abundant compared to the control in five or all cultivars. The amino acid threonine was strongly reduced (M202) or higher accumulated (Nipponbare, IR62266-42-6-2, IR57311) compared to the control.

**Figure 4:**
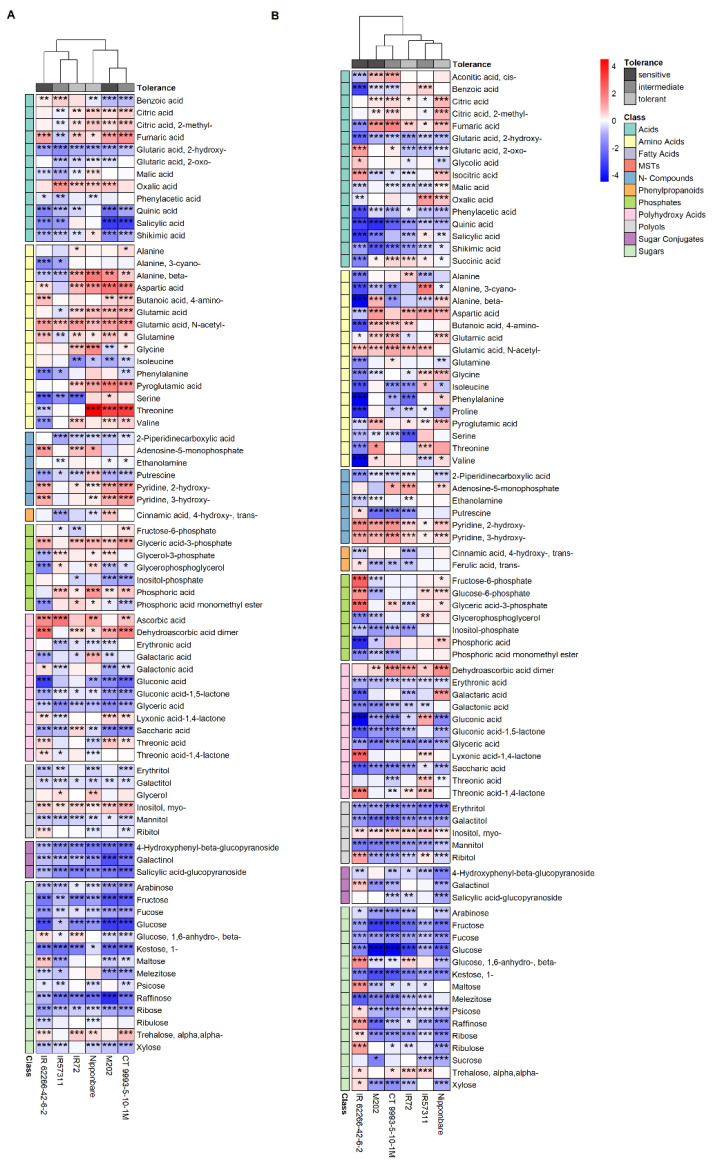
Changes in metabolite levels in leaf middle (**A**) and tip (**B**) segments represented as log_2_ fold changes. Red color indicates a higher log_2_ fold change, blue color a lower log_2_ fold change compared to the control. Asterisks display significance level of *p* values: <0.001—***, ≥0.001 and ≤0.01—**, ≥0.01 and ≤0.05—*. Tolerance and metabolite classes are color-coded. For a better overview, only metabolites with significant changes in at least two cultivars are shown.

The log_2_ fold changes in the metabolites present in leaf middle segments, clustered within the two subspecies *indica* and *japonica* (Figure 4A). Sugars and sugar conjugates showed lower metabolite abundances under HNT compared to control conditions. Again, the amino acid threonine was significantly higher accumulated under stress conditions, but only in *japonica* cultivars. Additionally, the log_2_ fold change in salicylic acid, galactonic acid and melezitose was significantly reduced in the intermediate and sensitive cultivars while for the tolerant ones, no significant change was detected.

Changes in the metabolite profiles of leaf tip segments were mainly grouped by tolerance classes, with the two sensitive cultivars and the intermediate CT9993-5-10-1M clustering together as well as the two tolerant cultivars and the intermediate IR57311 (Figure 4B). The acids cis-aconitic acid, benzoic acid and 2-oxo-glutaric acid, as well as the phosphate phosphoric acid monomethyl ester, had significantly different log_2_ fold changes in the first cultivar group compared to the second group. Polyols (erythritol, galactitol, mannitol) and sugars (arabinose, fructose, fucose, glucose, 1-kestose, melezitose) showed a lower accumulation under HNT compared to control conditions for five or all six cultivars. This was also observed for the sugars psicose, raffinose and ribose, with the exception of a higher log_2_ fold change in the sensitive cultivar IR62266-42-6-2.

**Figure 5:**
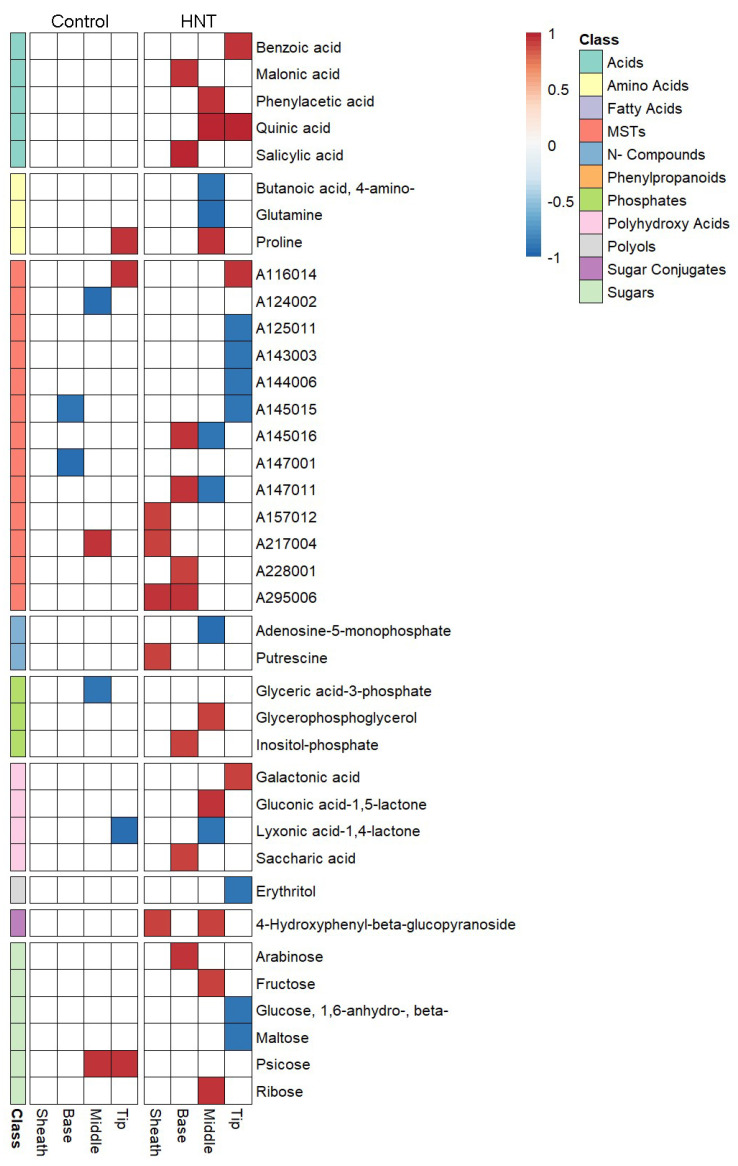
Heatmap of significant Spearman′s rank correlations (*p* < 0.05) between filtered metabolites per segment and the HNT sensitivity rank of the cultivars based on average chlorosis mean ranks.

To identify metabolites significantly related to HNT sensitivity, a correlation analysis was performed between the corresponding metabolites of each segment and the HNT sensitivity expressed as chlorosis rank (Figure 5). For this analysis, metabolites with missing values were excluded, resulting in 102 up to 136 metabolites, depending on segment and condition (Table 1).

HNT sensitivity in this study is based on chlorosis estimates with high ranks representing higher sensitivity to HNT stress. Therefore, a positive correlation between the metabolite pool size and the chlorosis rank refers to a high metabolite level in sensitive cultivars and a low level in tolerant cultivars, while a negative correlation reflects the opposite relationship.

For control conditions, five positive (proline, two unknown analytes and psicose) and five negative (three unknown analytes, glyceric acid-3-phosphate and lyxonic acid-1,4-lactone) significant correlations were identified in three out of four segments. Five of these correlations were lost under HNT conditions (two unknowns, acid-3-phosphate, 2x psicose). For HNT conditions, 26 positive and 13 negative correlations were identified. Metabolites of the tip segment comprised the highest number of negative correlations with HNT sensitivity, including four unknown analytes, the polyol erythritol and the sugars beta-1,6-anhydro-glucose and maltose. Metabolites of the middle segment with negative correlations with HNT sensitivity included two amino acids (4-amino-butanoic acid, glutamine) and two unknown analytes, the N-compound adenosine-5-monophosphate and lyxonic acid-1,4-lactone, whereas the segments sheath and base did not show any negative correlations. Positive correlations of metabolite abundances with HNT sensitivity were observed for all segments including acids (six in total, such as quinic acid or malonic acid), one amino acid (proline), unknown analytes (eight), the N-compound putrescine, two phosphates (glycerophosphoglycerol), the polyhydroxy acid saccharic acid, as well as sugars (arabinose, fructose, ribose) or sugar conjugates (4-hydroxyphenyl-beta-glucopyranoside).

### 2.4. Gene Expression in the Middle Part of the Leaf and the Sheath of Sensitive Cultivars Is Most Highly Affected by HNT Conditions

In addition to metabolite profiles, gene expression patterns of the leaf segments were analyzed under HNT compared to control conditions. Tolerance classes were reduced for this analysis into a sensitive (M202, CT9993-5-10-1M, IR62266-42-6-2) and a tolerant (IR57311, IR72, Nipponbare) group, following the results of the chlorosis ranking in this study and the metabolite clustering in leaf tips, the most affected leaf segment by chlorosis. RNA sequencing (RNA-Seq) was performed for each cultivar, condition and segment in one replicate. Libraries contained between 22,747,286 and 49,046,226 single-end reads, and between 93.9% and 99.6% of these reads were aligned to the Nipponbare reference genome (Supplementary Table S1).

**Figure 6:**
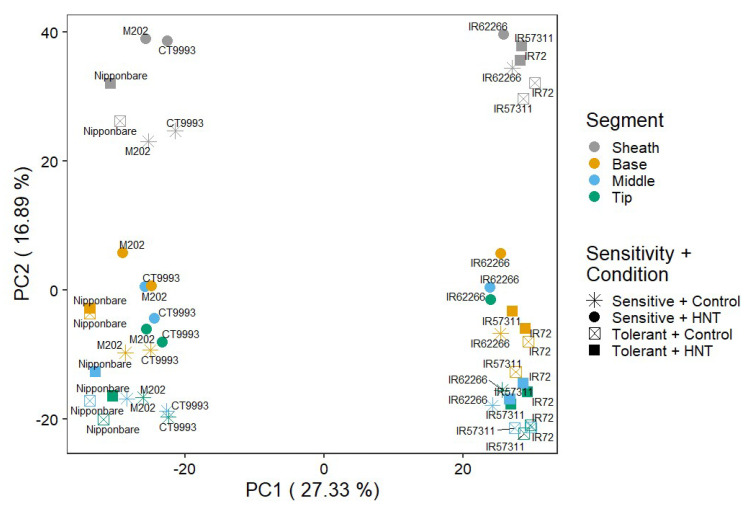
Principle component analysis of RNA-Seq raw counts from samples of all four rice leaf segments exposed to control and HNT conditions.

In contrast to the metabolite profiling, a global analysis of the count distribution over all samples revealed only a clear separation between sheath and the remaining leaf segments by PC2 (Figure 6). That may derive from the high variance between the replicate samples representing each a different rice cultivar in this analysis. However, a clear separation between cultivars of the *O. sativa* subspecies *indica* and *japonica* was observed by PC1, explaining 27.33% of the variance.

**Figure 7:**
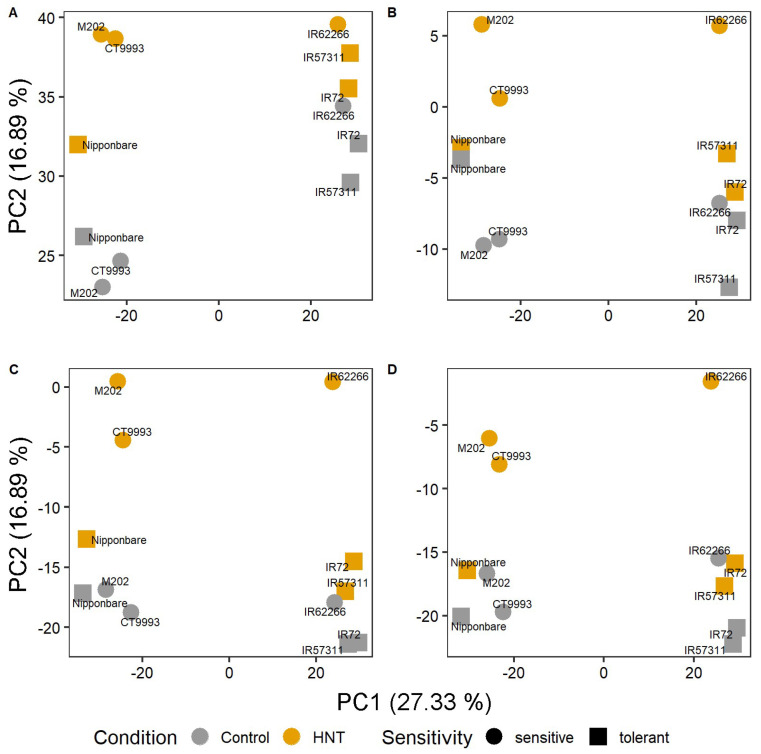
Principle component analysis of RNA-Seq raw counts from leaf segments samples exposed to control and HNT conditions separated for sheath (**A**), base (**B**), middle (**C**) and tip (**D**). PCA is as in Figure 6, with HNT and control color-coded and HNT sensitivity of cultivars marked with specific symbols.

Investigating the differences per segment in detail, a clearer separation between control and HNT conditions in addition to the subspecies effect was observed for all segments, with sensitive cultivars showing a higher variance compared to tolerant ones (Figure 7). A higher variance of count distribution was determined for middle and tip segments at HNT compared to control conditions and RNA-Seq data of HNT-tolerant cultivars scattered closer together compared to HNT-sensitive ones (Figure 7C,D). The HNT samples of the HNT-sensitive cultivars M202, CT9993-5-10-1M and IR62266-42-6-2 were in all leaf segments separated from all other samples by PC2, whereas HNT and control samples from HNT-tolerant cultivars clustered together with control samples from HNT-sensitive cultivars.

Differential genes expression (DGE) analysis was performed using the R-packages DESeq2 and edgeR. Due to the limited number of samples, both approaches were performed and the overlapping significant differently expressed genes (DEGs) between both analyses used for further analysis (Table 2). In general, more significant DEGs were obtained for the HNT-sensitive compared to the HNT-tolerant group. For the sensitive group, between 2 (base) and 78 (middle) DEGs were determined (Supplementary Table S2), while for the tolerant group, the numbers of DEGs ranged between one (base) and ten (middle) (Supplementary Table S3). Most DEGs were detected in the middle part of the leaves, followed by sheaths and tips. Transcript expression in the base of the leaf was almost not affected by HNT stress conditions.

### 2.5. Identification of Segment Specific Differentially Expressed Genes Regulating HNT Response

**Figure 8:**
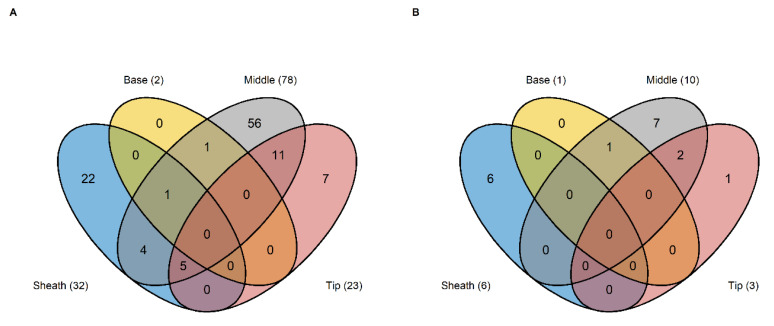
Venn plots representing overlapping significant differentially expressed genes for all leaf segments of the HNT-sensitive (**A**) and HNT-tolerant (**B**) cultivar group.

For a better understanding of specific gene regulation per segment, uniquely expressed genes specific for each segment were identified for the HNT-sensitive (Figure 8A) and HNT-tolerant group (Figure 8B).

For both tolerance classes, no common overlap of DEGs between the four segments was determined. Within the sensitive group, most of the uniquely expressed genes were identified for the leaf middle segment with 56 DEGs followed by the sheath and the tip segment with 22 and seven unique DEGs (Figure 8A). Within the tolerant group, the highest number of seven unique DEGs were also identified for the leaf middle segment (Figure 8B). However, only a small number of DEGs was identified for this group in sheath or tip segments with only six or one uniquely expressed DEGs.

Uniquely expressed genes per segment were extracted for both groups and interesting candidates selected which may be linked to the HNT stress response (Table 3). For the complete gene list, see Supplementary Table S4 for the HNT-sensitive group and Supplementary Table S5 for the HNT-tolerant group.

The overlap of DEGs between this study and a previous transcriptomic study of whole rice leaves under HNT [22] was determined. The previous study excluded leaf sheaths and was based on an analysis with a microarray platform. Similar to our study, more DEGs were obtained for sensitive compared to tolerant cultivars (Table 4).

For cultivars of the tolerant group, no overlapping DEGs between the two studies were identified by comparing low numbers of 10 against 29 DEGs. An overlap of 36 unique DEGs between whole leaves (former study) and the three leaf blade segments, base, middle and tip (this study), was identified for the HNT-sensitive group, comparing in total 85 against 550 DEGs (Supplementary Table S5).

In total, 13 DEGs were identified overlapping in at least two out of three sensitive cultivars included in the previous microarray analysis and in the sensitive group of the new RNA-Seq analysis, indicating an important role during HNT response in rice (Table 5). Those genes encoded, for example, a heat stress transcription factor *HSFB1*, a Bowman–Birk family proteinase inhibitor *RBBBI3-2*, a TIFY domain containing protein *OsTIFY11d* or an alpha- and beta-expansin (*OsEXP2*, *OsEXPB3*). For a complete list of all DEGs overlapping with at least one sensitive cultivar of the microarray analysis [22] and a significant expression either for edgeR and/or DESeq2, see Supplementary Table S6.

## 3. Discussion

### 3.1. Higher Raffinose Abundance in the Sheath Compared to Leaf Blade Segments Reveals a Pre-Adaptation to Abiotic Stress under Control Conditions

The metabolic and transcriptomic characterization of the leaf sheath and leaf blade segments under control conditions was the pre-requisite to investigate changes caused by HNT stress. The sheath at the leaf basis is important for protection of the inner leaf and for stabilization of the leaf blade [25]. In addition to contributing to the robustness of the leaf, it is a site of various metabolic and regulatory processes [26]. Only a few publications investigated metabolite composition of the sheath so far, mainly for research on sheath development [25,27], in the context of biotic stress responses to sheath blight (reviewed in [28]) or for characterization of bacterial communities in leaf sheaths versus leaf blades [29]. To the best of our knowledge, no study exists on the comparison of leaf sheath metabolite composition in different cultivars compared to other segments of the leaf blade or under HNT conditions. Under control conditions, the leaf sheath was clearly different from all other segments, indicated by the lowest number of annotated metabolites and a general lower abundance of main primary metabolites. A study investigating the developmental mechanisms of leaf sheaths, blades and the blade–sheath boundary region using microarray analysis reported huge transcriptome differences between them at the mature stage [25], which might be also reflected by metabolic differences.

In a transcriptomic and metabolic study of different organs during grain development in rice cultivar Zhongua11, samples of sheath and leaf at the booting stage clustered together and were separated from the other organs [27]. Nevertheless, a direct comparison of leaf sheath and leaf blade highlighted that metabolic processes such as photosynthesis, photorespiration and fatty acid biosynthesis were more pronounced in leaves as the main photosynthesis organ compared to sheaths [27]. A lower total metabolite concentration in the sheath of *Oryza sativa* L. cv. Koshihikari was also observed in a study on bacterial communities [29]. Contrarily, in this study, the authors found higher values for sugars such as glucose, fructose and sucrose, which were not confirmed by our study. Leaf sheaths were described to function as carbohydrate sinks before heading [30], also shown by maximum starch accumulation during panicle development in the sheath [31]. Underlining this finding, genes encoding enzymes of carbohydrate metabolism were enriched in mature sheath tissue [25]. Differences in carbohydrate detection in our study compared to other ones might be based on the different age of plants, as leaves in our study were still in the vegetative stage.

Interestingly, in sheaths of all investigated cultivars, a higher accumulation of raffinose and its precursor galactinol, both substances well known to be accumulated under various stress conditions [32], point to a pre-adaptation to stress conditions in this organ. In accordance, also the gene expression of related genes as galactinol synthase 1 (*OsGolS1*, Os03g0316200), galactinol synthase 2 (*OsGolS2*, Os07g0687900) and raffinose synthase (*OsRf5*, Os01g0170000) averaged over the sheaths of all cultivars was 6.9-fold, 1.8-fold and 3.1-fold higher than averaged over all other segments and cultivars under control conditions. Growth of grass leaves occurs at the basal part called the growth zone [33], identifying the leaf base as most protection deserving structure. As the sheath protects the leaf base from temperature fluctuations and water limitations, an accumulation of osmoprotectants already under control conditions supports a fast response to upcoming stress events and secures the survival of the basal growth zone during severe stress as long as possible. Importance of the sheath in the response to environmental stresses was also suggested when an overrepresented gene expression in a protein kinase GO term, involved in signaling, was found in mature sheath tissue [25]. Even bacterial community structures of leaf sheath associated bacteria were more tolerant to environmental factors compared to communities of the leaf blade [29], suggesting again that the sheath is the site of highest stress exposure with the need for an immediate response. If higher raffinose abundances found in *indica* cultivars in our study are able to contribute to higher resistance levels against sheath blight reported for *indica* cultivars [28], this must be proven.

### 3.2. The Leaf Sheath Contributes to HNT Response on Transcriptomic Level

Under HNT conditions, a large group of metabolites present in the sheath were still less abundant than in segments of the leaf blade (not shown). For changes in metabolite levels under HNT in the sheath, very few correlations with HNT sensitivity were found. The correlation analysis only revealed putrescine, 4-hydroxyphenyl-beta-glucopyranoside and three unknown analytes as correlated with HNT sensitivity in sheaths. Higher polyamine levels (putrescine, spermidine and spermine) were previously reported for leaf blades of sensitive cultivars when investigating 12 cultivars with contrasting HNT sensitivity [21]. Simultaneously, expression of arginine decarboxylase 2 (*ADC2*) and ornithine decarboxylase (*ODC1*) encoding enzymes catalyzing the first committed step of putrescine biosynthesis were increased only in sensitive cultivars [21].

**Figure 9:**
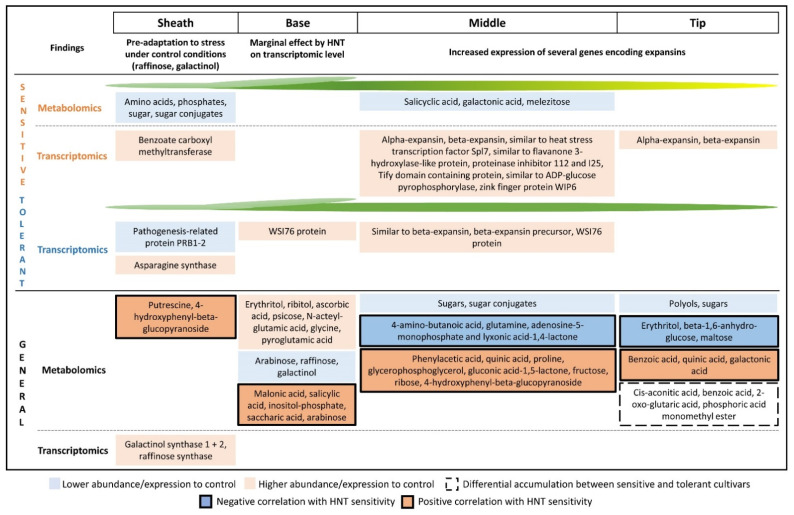
Overview of main metabolic and transcriptomic changes in the leaf sheath and segments of the leaf blade in sensitive or tolerant cultivars under HNT conditions. General changes under HNT in all investigated cultivars are shown at the bottom of the figure.

More striking were the significant transcriptomic changes in the sheaths of the investigated cultivars in response to HNT. Six differentially expressed transcripts were found in sheaths of the three tolerant cultivars, with a 5.3-fold increased expression of asparagine synthetase being the most mentionable. Higher expression of this gene in the tolerant group contradicts previous findings on high asparagine levels as metabolite marker for HNT sensitivity [21,22], except that all previous studies investigated whole leaf blades. On the other hand, asparagine was found to be increased in panicles of rice cultivars in HNT field experiments in the dry season and was related to a successful detoxification of cyanide via 3-cyano-alanine [34]. An overview about the metabolic and transcriptomic differences in all leaf segments is given in Figure 9.

### 3.3. The Leaf Base Was Marginally Affected by HNT, Especially on Transcriptomic Level

The general pattern of metabolite changes in the leaf base did not show any clustering according to the HNT tolerance class or subspecies of the cultivar and seemed to be rather genotype-specific. Nevertheless, the abundance of nine metabolites present in the leaf base was positively correlated with HNT sensitivity, with higher abundances in more HNT-sensitive cultivars (Figure 9). In our previous study, five of these metabolites were also found to be positively correlated with HNT sensitivity when whole leaf blades of the same cultivars were considered, including salicylic acid, saccharic acid, arabinose and two unknown analytes, A228001 and A295006 [22]. These metabolic changes were not reflected by transcriptomic changes as only one or two significant differentially expressed genes were found in the leaf base of the tolerant or sensitive cultivar group. This finding points to a transport of these metabolites to the base under HNT conditions rather than a *de novo* synthesis driven by increased gene expression.

One differentially higher expressed gene was found in the leaf base of tolerant cultivars, the WSI76 protein induced by water stress (Os07g0687900), also annotated as galactinol synthase (e.g., rice annotation project database—RAP-DB) (Supplementary Table S3), suggesting a possible water stress response linked to HNT. In addition, this transcript was significantly induced in tolerant cultivars in the middle part (Supplementary Table S3, middle). This enzyme catalyzes the synthesis of galactinol from inositol and UDP galactose, which then is the substrate for raffinose biosynthesis. Galactinol synthases have been described previously in response to abiotic stress in rice [35,36] and the overexpression of *AtGolS2* in rice contributed to increased drought tolerance and yield [37]. An impact of HNT on daytime water use was recently suggested during the seedling stage for wheat due to higher transpiration rates during the day [38]. HNT-tolerant cultivars might overcome an imminent vapor pressure deficit, which is supposed to reduce water and carbon availability during HNT [38] in the base by inducing this galactinol synthase.

No overlap of significant differentially expressed transcripts in the leaf base was found with our previous transcriptomic study of the whole leaf blade under HNT conditions in the same cultivars [22], suggesting that the HNT responsive transcriptomic changes reported in that study could be only detected when considering expression changes in the whole leaf.

### 3.4. The Middle Part and the Tip of the Leaf Are Most Highly Affected by HNT on Metabolic and Transcriptomic Level

The middle part and the tip of the leaf are the segments, which were most highly affected by chlorosis and necrosis and displayed the highest number of significantly changed metabolites under HNT conditions. For both segments, the highest number of correlations of metabolite abundance with HNT sensitivity was found under stress conditions (14 for the middle and 11 for the tip). Only a significantly positive correlation between fructose abundances and HNT sensitivity was confirmed from our previous study of whole leaf blades with the same cultivars [22]. Furthermore, only two positive correlation of proline and glycerophosphoglycerol with HNT sensitivity were confirmed in a study with 12 rice cultivars [21], suggesting a segment-specific metabolic HNT response not only for the sheath and base but also for the middle part and the tip (Figure 9).

In the middle part, an especially high number of significantly expressed transcripts was detected, with 13 of them overlapping with transcriptomic changes in leaf blades of sensitive cultivars under HNT in the previous study [22]. This specific HNT response for sensitive cultivars in the middle part and partly in the tip included genes encoding proteins involved in regulation, e.g., a protein similar to heat stress transcription factor Spl7 (Os09g0456800), a zinc finger protein WIP6 (Os05g0444200) or a proteinase inhibitor 112 of the Bowman–Birk family (Os01g0124100). Bowman–Birk inhibitors (BBIs) are serine-type protease inhibitors important for plant biotic defense mechanisms against phytophagous insects and fungal and bacterial pathogens [39]. They are also described to play a role in abiotic stress responses to drought, heat, salt, oxidative stress and Fe-deficiency (for reviews, see [39,40,41].

Another regulatory protein encoded by a gene expressed in the middle leaf of sensitive cultivars was a Tify domain containing protein (synonyms OsTIFY11d, OsJAz1, OsJAZ12) (Os10g0392400), which was described in rice as a transcriptional regulator within the jasmonate-signaling related BHLH148-TIFY11D-COI1A complex conferring drought tolerance in rice [42]. The finding of higher expression of this gene underlines the importance of jasmonate signaling in HNT response suggested previously [22].

Furthermore, a gene encoding an enzyme similar to a flavanone-3-hydroxylase-like protein was significantly enhanced under HNT in sensitive cultivars in both studies. This enzyme is a key enzyme in flavonoid biosynthesis conferring 3′-hydroxylation of flavonoids [43], suggesting an up-regulation of the biosynthesis of secondary metabolites in sensitive cultivars. An up-regulation of phenylalanine ammonia-lyase (PAL) catalyzing the first step in the phenylpropanoid biosynthesis was previously found for sensitive as well as tolerant cultivars in response to HNT [22].

Remarkable was the high number of differentially expressed transcripts encoding expansins or expansin precursor proteins under HNT conditions in the middle part and partly the tip of sensitive cultivars. Four of these genes were under the nine most highly expressed genes in the middle part with a log_2_ fold change above five: Alpha-expansin (*OsEXPA4*, Os05g0477600), Beta-expansin precursor (*OsExpB6*, Os10g0555600), Beta-expansin (*OsExpB11*, Os02g0658800) and Similar to Beta-expansin (*OsEXPB4*, Os10g0556100). In addition, alpha-expansin (*OsEXPA2*, Os01g0823100) and Similar to Beta-expansin (*OsExpB3*, Os10g0555900) were significantly highly differentially expressed in both the middle and tips of sensitive cultivars. Furthermore, four of these genes were also expressed uniquely in the middle leaf of tolerant cultivars: Alpha-expansin (*OsEXPA4*), Beta-expansin precursor (*OsExpB6*), Alpha-expansin (*OsEXPA2*) and Similar to Beta-expansin (*OsExpB3*), all from either the α-expansin (EXPA) or β-expansin (EXPB) subfamily.

Expansins have cell wall loosening functions, contribute to cell expansion and are involved in numerous physiological and developmental processes. They are further described to be involved in multiple abiotic stress responses and in improved nutrient uptake (for reviews, see [44,45]). Stress responses closely related to HNT stress are water and heat stress conditions. In *Crateostigma plantagineum*, expansins were strongly affected by water deficiency [46] and the overexpression of a wheat expansin successfully improved drought tolerance in tobacco [47]. Expansins were in addition upregulated in response to heat stress in grasses [48] and tobacco plants were more heat tolerant when an expansin gene from grasses was overexpressed [49]. Overexpression of selected expansins in cereals improved abiotic stress tolerance to salt stress in rice [50] and to drought in wheat [51]. However, their exact function is still not clear. Overexpression of expansins influenced the oxidative stress response, specifically the cell wall-bound peroxidase activity [52], and changed the cell structure, resulting in shorter stems and curly leaves and leaf blades being more compact with a lower number of stomata [53]. Tissue-specific expression was reported for different expansins in wheat [54], which might explain the almost exclusive finding as differentially expressed transcripts in the middle part and the tip. The distinct function of expansins in HNT stress response remains to be elucidated.

## 4. Material and Methods

### 4.1. Plant Material, Cultivation and Experimental Design of HNT Stress Treatment

For the analysis, six rice cultivars from the subspecies *indica* and *japonica* were selected with different tolerances to high night temperature (HNT) stress [15] grouped into sensitive (IR62266-42-62, M202), intermediate (IR57311, CT9993-5-10-1M) and tolerant (IR72, Nipponbare) classes. Experiments were performed in a controlled climate chamber with a 12 h light regime with 70% relative humidity and a temperature setting of 26 °C in the light and 22 °C at night for control conditions, or 28 °C constant in the light and at night (HNT). Seed germination, planting and stress treatment were performed as previously described [15].

For phenotyping, fifteen plants per cultivar were characterized in the vegetative stage, before the start of the HNT-treatment (25 days after sowing (DAS) and 21 days after treatment (46 DAS)) under HNT or control conditions by measuring leaf length and tiller number and performing a visual scoring of chlorosis and necrosis of all leaves, as previously described [15]. Scoring was based on a pre-defined scale and resulting scores were ranked by Excel’s in-built function RANK.EQ. Averages of the ranks were calculated to represent different levels of damage. For the identification of significant differences, a Kruskal–Wallis test was performed using the Kruskal function including a multiple error correction (“BH”) from the R-package agricolae v1.3.3 (https://cran.r-project.org/web/packages/agricolae/index.html, accessed on 1 June 2021).

**Figure 10:**
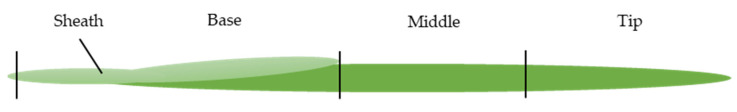
Schematic representation of rice leaf segmentation for analysis. Leaves were divided into sheath, base, middle and tip segments.

For omics measurements, samples of leaves of the same age were taken 46 DAS around 4 to 6 h under light at control and HNT conditions and immediately frozen in liquid nitrogen. Each leaf was divided into four parts, leaf sheath, base and the potentially damaged middle part and tip (Figure 10).

### 4.2. Metabolite Profiling and Data Analysis

Metabolite profiling was performed by gas chromatography coupled to electron impact ionization time of flight mass spectrometry (GC/EI-TOF-MS), as described previously [55]. For analysis, fractions enriched in polar primary metabolites were extracted from 120 mg of grounded leaf material for each sample, as described before [56]. Five replicates were measured per cultivar, condition and leaf segment 21 days after treatment initiation (46 DAS) from the same plants used for phenotypic analyses. Initial raw intensities were normalized by internal standard and approximate dry weight per sample. Approximate dry weight was obtained by using the ratio of fresh weight to dry weight for three random replicates per cultivar, condition and segment. The dry weight itself was determined by freeze-drying the grinded leaf material in Eppendorf tubes and the weight was recorded before and after drying.

In total, 230 primary metabolites were annotated (Supplementary Table S7). Metabolite raw data are available at MetaboLights [57] (https://www.ebi.ac.uk/metabolights/, accessed on 2 July 2021) under the accession number MTBLS2991. For the individual segments, 206 (sheath), 212 (base), 224 (middle) and 228 (tip) metabolites were annotated. Metabolites with more than 25% missing values over all samples and known contaminations, annotated as reagents and internal standards, were removed, resulting in 137 metabolites for further analysis. Missing values for the remaining metabolites were imputed by half the minimum value per metabolite over all samples. For the filtered and imputed data, each metabolite was divided by the median of the respective metabolite over all samples and data were log_2_ transformed afterwards. Finally, averages of transformed data were calculated per condition, segment and cultivar, and log_2_ fold changes were obtained and *p* values determined by Fisher’s exact test. The significance threshold was set to a false discovery rate of <0.05. Correlation analysis was performed with log_2_ median transformed data and the chlorosis mean rank utilizing the R function cor.test with the Spearman method and a significance threshold of <0.05.

### 4.3. Transcriptome Profiling

For transcriptome profiling, homogenized leaf material of five replicates per cultivar, condition and segment was pooled to 100 mg, resulting in 48 samples (one sample per cultivar, condition and segment). Samples were the same as for metabolite profiling and taken after 21 days of HNT treatment initiation (46 DAS). Total RNA was isolated using a TRIzol protocol based on the “single step” method [58]. Then, 4 µg of each RNA sample were treated with DNase (RapidOut DNA-removal Kit, Thermo Scientific) and the absence of genomic DNA contamination was confirmed by qRT–PCR using intron-specific primers [59]. RNA quality and integrity were verified with the Agilent 2100 Bioanalyzer (Agilent Technologies, Santa Clara, CA, USA).

Library preparation and RNA sequencing were performed at the Max Planck Genome Center Cologne, Germany (https://mpgc.mpipz.mpg.de/home, accessed on 15 August 2021). Sample libraries were prepared with the NEBNext Ultra Directional RNA Library Prep Kit for Illumina (New England Biolabs). Sequencing was performed on an Illumina HiSeq 3000 platform, resulting in 100 base pair (bp) single-end reads. RNA-Seq raw data are available at GEO (http://www.ncbi.nlm.nih.gov/geo, accessed on 7 July 2021) [60] under the accession number GSE179662.

### 4.4. RNA-Seq Data Analysis

Raw read quality per library was checked with FastQC v0.11.8 [61]. To avoid multi-mapping, reads shorter than 80 bp were discarded and adapter trimmed, both steps using trimmomatic v0.36 [62]. To obtain three samples for further downstream analysis, tolerance classes were reduced to only two, HNT-sensitive (cultivars IR62266-42-6-2, M202, CT9993-5-10-1M) and HNT-tolerant (cultivars IR57311, IR72, Nipponbare), based the results of the chlorosis ranking in this study and the metabolite clustering in leaf tips, the most affected leaf segment by chlorosis. Read mapping was performed with STAR 2.7.1a [63] with default parameters against the genome sequence of *Oryza sativa* ssp. japonica, cultivar Nipponbare, release IRGSP-1.0.49 (downloaded from https://plants.ensembl.org/info/data/ftp/index.html, accessed on 17 December 2020).

Differential gene expression (DGE) analysis was performed with DESeq2 [64] and edgeR [65] with default settings. Resulting *p* values were corrected for multiple testing errors [66]. The cut-off for DGE was set to a false discovery rate < 0.1 for DESeq2 and <0.05 for edgeR; for both approaches, an absolute log_2_ fold change of ≥1 was used. For further analysis, the overlap of significantly differently expressed genes (DEGs) between both methods was used.

### 4.5. Data Visualization

Data analysis and figure generation were performed in R v4.0.3 [67] and RStudio v 1.4.1103 [68]. Principle component analysis for metabolite data was calculated with pcaMethods v1.82.0 [69] using pareto scaling and centering. For transcriptome data, DESeq2 was utilized to calculate variances for PCA with the vst-function. PCA- and bar plots were visualized with ggplot2 v3.3.3 [70]. Heatmaps were generated with the package pheatmap v1.0.12 (https://cran.r-project.org/package=pheatmap, accessed on 15 May 2021). For Venn plots, the package ggvenn v0.1.8 (https://cran.r-project.org/web/packages/ggvenn/index.html, accessed on 15 May 2021) was used.

## 5. Conclusions

A differentiated response of three leaf blade segments and leaf sheath was discovered on metabolic and transcriptomic level in six rice cultivars with contrasting HNT tolerance. Stress pre-adaptation by higher raffinose levels in the sheath was discovered under control conditions together with a lower abundance for larger groups of metabolites compared to the leaf blade. Only very few molecular changes contributed to the HNT response in the sheath. Correlations of some metabolites with HNT sensitivity from a previous study could be confirmed for the base. However, metabolic changes in the leaf base were not reflected by transcriptomic changes. Increased expression levels of galactinol synthase in the base suggested that HNT might elicit slight water stress effects. Transcriptomic changes in the middle part and the tip have driven the HNT response in vegetative rice leaves. HNT transcriptomic response in the middle part of the leaf blade included regulatory processes, jasmonic acid related regulation, biosynthesis of flavonoids and remarkable involvement of expansins in the HNT response.

Investigating the differential response of leaf segments and the sheath suggested segment-specific HNT responses for sensitive and tolerant cultivars and emphasized the leaf base as a stress protected area with the lowest number of transcriptomic changes.

## Figures and Tables

**Table 1:** Number of metabolites after filtering of metabolites with missing values for correlation analysis per segment and condition.

Condition	Sheath	Base	Middle	Tip
Control	102	130	136	136
HNT	108	129	134	133

**Table 2:** Overlap of significant differentially expressed genes between the approaches DESeq2 (FDR < 0.1; |log2FC| ≥ 1) and edgeR (FDR < 0.05; |log2FC| ≥ 1) for rice leaf segments upon HNT stress. Three cultivars each were analyzed for the sensitive (M202, CT9993-5-10-1M, IR62266-42-6-2) and the tolerant (IR67311, IR72, Nipponbare) group.

Tolerance Group	Segment	Up	Down	Total
Sensitive	Sheath	20	12	32
	Base	0	2	2
	Middle	54	24	78
	Tip	8	15	23
Tolerant	Sheath	3	3	6
	Base	1	0	1
	Middle	9	1	10
	Tip	2	1	3

**Table 3:** Selected unique differentially expressed genes per segment with an absolute log_2_ fold change larger than five for HNT-sensitive and HNT-tolerant rice cultivars. “+”, up-regulated; “-”, down-regulated.

Group	Tissue	Gene Identifier	Description	Up/Down
Sensitive	Sheath	Os11g0147150	Hypothetical gene	+
		Os08g0287200	Hypothetical conserved gene	+
		Os06g0242000	Similar to benzoate carboxyl methyltransferase	+
	Middle	Os12g0282000	Conserved hypothetical protein	-
		Os05g0477600	Alpha-expansin OsEXPA4	+
		Os04g0344100	Similar to OSIGBa0106G08.3 protein	+
		Os10g0555600	Beta-expansin precursor	+
		Os02g0658800	Beta-expansin	+
		Os04g0418800	Similar to Hydroxyproline-rich glycoprotein	+
		Os10g0556100	Similar to beta-expansin EXPB4	+
		Os04g0350100	Proteinase inhibitor I25, cystatin domain containing protein	+
		Os02g0236600	Peroxidase P7 (EC 1.11.1.7) (TP7)	+
		Os02g0112900	Similar to Viroid RNA-binding protein (Fragment)	+
	Tip	Os04g0493600	Similar to Lectin-C precursor (PL-C)	+
Tolerant	Sheath	Os01g0382000	Similar to Pathogenesis-related protein PRB1-2 precursor	-
		Os03g0291500	Asparagine synthetase	+
		Os01g0159000	Similar to cDNA clone: J023049H21	+
		Os01g0550800	Protein of unknown function DUF239, plant domain containing protein	+
	Middle	Os10g0555900	Similar to Beta-expansin	+
		Os10g0555600	Beta-expansin precursor	+

**Table 4:** Assessment of significant differentially expressed genes (DEGs) from the RNA-Seq analysis of leaf blade segments (base, middle, tip) and a whole leaf microarray analysis [22] for sensitive (M202, CT9993-5-10-1M, IR62266-42-6-2) and tolerant cultivars (IR57331, IR72, Nipponbare).

	#DEGs RNA-Seq	#DEGs Glaubitz et al. 2017	Overlap
Sensitive	85	550	36
Tolerant	10	29	0

**Table 5:** Overlap of significant differentially expressed genes and respective log_2_ fold changes in at least two out of three HNT-sensitive rice cultivars included in a whole leaf microarray analysis (CT9993, M202, IR62266) [22] and in the HNT-sensitive group used for the RNA-Seq analysis in this study (edgeR and DESeq2) including the leaf blade segments base, middle and tip.

ID	Segment	Function	CT9993	M202	IR62266	edgeR	DESeq2
Os04g0689500	Middle	Conserved hypothetical protein.	NA	3.87	3.59	2.80	2.80
Os09g0456800	Middle	Similar to Heat stress transcription factor Spl7 (Heat shock transcription factor).	NA	4.88	3.60	3.37	3.37
Os05g0444200	Middle	Similar to T6J4.5 protein (WIP6 protein).	4.60	5.80	5.50	3.43	3.42
Os04g0581000	Middle	Similar to Flavanone 3-hydroxylase-like protein.	NA	8.32	3.83	3.55	3.55
Os09g0272600	Middle	Conserved hypothetical protein.	NA	3.67	4.48	3.97	3.96
Os01g0124100	Middle	Proteinase inhibitor I12, Bowman–Birk family protein.	NA	3.73	5.24	4.05	4.07
Os05g0550300	Middle	Similar to Lipid transfer protein (Fragment).	4.31	4.64	4.40	4.14	4.14
Os10g0392400	Middle	Tify domain containing protein.	5.02	5.20	6.08	4.16	4.15
Os05g0580000	Middle, Tip	Similar to ADP-glucose pyrophosphorylase (EC 2.7.7.27).	NA	4.29	7.41	4.65	4.64
Os02g0106100	Middle	Similar to Fructosyltransferase.	3.99	4.97	3.67	4.70	4.69
Os01g0823100	Middle, Tip	Alpha-expansin OsEXPA2.	NA	5.61	4.36	4.84	4.85
Os10g0555900	Middle, Tip	Beta-expansin precursor.	NA	6.70	4.36	5.96	5.95
Os04g0659300	Middle, Tip	Protein of unknown function DUF26 domain containing protein.	NA	6.34	6.58	7.58	7.65

## Data Availability

The data presented in this study are available in the Supplementary Materials and at GEO (http://www.ncbi.nlm.nih.gov/geo accessed on 22 September 2021) under the accession number GSE179662 or at MetaboLights (https://www.ebi.ac.uk/metabolights/ accessed on 22 September 2021) under the accession number MTBLS2991.

## Supplementary Materials

The following are available online at https://www.mdpi.com/article/10.3390/ijms221910451/s1.

## References

[1] Wassmann R., Jagadish S.V.K., Sumfleth K., Pathak H., Howell G., Ismail A., Serraj R., Redona E., Singh R.K., Heuer S. (2009). Regional vulnerability of climate change impacts on Asian rice production and scope for adaptation. Advances in Agronomy.

[2] Muthayya S., Sugimoto J.D., Montgomery S., Maberly G.F. (2014). An overview of global rice production, supply, trade, and consumption. Ann. N. Y. Acad. Sci..

[3] IPCC Global Warming of 1.5 °C. An IPCC Special Report on the Impacts of Global Warming of 1.5 °C above Pre-Industrial Levels and Related Global Greenhouse Gas Emission Pathways, in the Context of Strengthening the Global Response to the Threat of Climate Change, Sustainable Development, and Efforts to Eradicate Poverty. https://www.ipcc.ch/sr15/.

[4] Pachauri R.K., Meyer L.A., IPCC, Core Writing Team (2014). Climate Change 2014: Synthesis Report. Contribution of Working Groups I, II and III to the Fifth Assessment Report of the Intergovernmental Panel on Climate Change.

[5] Easterling D.R., Horton B., Jones P.D., Peterson T.C., Karl T.R., Parker D.E., Salinger M.J., Razuvayev V., Plummer N., Jamason P. (1997). Maximum and minimum temperature trends for the globe. Science.

[6] Vose R.S., Easterling D.R., Gleason B. (2005). Maximum and minimum temperature trends for the globe: An update through 2004. Geophys. Res. Lett..

[7] Wang K., Li Y., Wang Y., Yang X. (2017). On the asymmetry of the urban daily air temperature cycle. J. Geophys. Res. Atmos..

[8] Peng S., Huang J., Sheehy J.E., Laza R.C., Visperas R.M., Zhong X., Centeno G.S., Khush G.S., Cassman K.G. (2004). Rice yields decline with higher night temperature from global warming. Proc. Natl. Acad. Sci. USA.

[9] Shi W., Muthurajan R., Rahman H., Selvam J., Peng S., Zou Y., Jagadish K.S. (2013). Source-sink dynamics and proteomic reprogramming under elevated night temperature and their impact on rice yield and grain quality. New Phytol..

[10] Bahuguna R.N., Solis C.A., Shi W., Jagadish K.S. (2017). Post-flowering night respiration and altered sink activity account for high night temperature-induced grain yield and quality loss in rice (*Oryza sativa* L.). Physiol. Plant..

[11] Impa S.M., Raju B., Hein N.T., Sandhu J., Prasad P.V.V., Walia H., Jagadish S.V.K. (2021). High night temperature effects on wheat and rice: Current status and way forward. Plant Cell Environ..

[12] Xu J., Misra G., Sreenivasulu N., Henry A. (2021). What happens at night? Physiological mechanisms related to maintaining grain yield under high night temperature in rice. Plant Cell Environ..

[13] Cooper N.T.W., Siebenmorgen T.J., Counce P.A. (2008). Effects of nighttime temperature during kernel development on rice physicochemical properties. Cereal Chem..

[14] Ambardekar A.A., Siebenmorgen T.J., Counce P.A., Lanning S.B., Mauromoustakos A. (2011). Impact of field-scale nighttime air temperatures during kernel development on rice milling quality. Field Crops Res..

[15] Glaubitz U., Li X., Köhl K.I., van Dongen J.T., Hincha D.K., Zuther E. (2014). Differential physiological responses of different rice (*Oryza sativa*) cultivars to elevated night temperature during vegetative growth. Funct. Plant Biol..

[16] Coast O., Ellis R.H., Murdoch A.J., Quiñones C., Jagadish K.S.V. (2015). High night temperature induces contrasting responses for spikelet fertility, spikelet tissue temperature, flowering characteristics and grain quality in rice. Funct. Plant Biol..

[17] Wu C., Cui K., Wang W., Li Q., Fahad S., Hu Q., Huang J., Nie L., Peng S. (2016). Heat-induced phytohormone changes are associated with disrupted early reproductive development and reduced yield in rice. Sci. Rep..

[18] Lanning S.B., Siebenmorgen T.J., Counce P.A., Ambardekar A.A., Mauromoustakos A. (2011). Extreme nighttime air temperatures in 2010 impact rice chalkiness and milling quality. Field Crops Res..

[19] Zhang Y., Tang Q., Peng S., Zou Y., Chen S., Shi W., Qin J., Laza M.R.C. (2013). Effects of high night temperature on yield and agronomic traits of irrigated rice under field chamber system conditions. Aust. J. Crop Sci..

[20] Shi W., Yin X., Struik P.C., Xie F., Schmidt R.C., Jagadish K.S.V. (2016). Grain yield and quality responses of tropical hybrid rice to high night-time temperature. Field Crops Res..

[21] Glaubitz U., Erban A., Kopka J., Hincha D.K., Zuther E. (2015). High night temperature strongly impacts TCA cycle, amino acid and polyamine biosynthetic pathways in rice in a sensitivity-dependent manner. J. Exp. Bot..

[22] Glaubitz U., Li X., Schaedel S., Erban A., Sulpice R., Kopka J., Hincha D.K., Zuther E. (2017). Integrated analysis of rice transcriptomic and metabolomic responses to elevated night temperatures identifies sensitivity- and tolerance-related profiles. Plant Cell Environ..

[23] Schaarschmidt S., Lawas L.M.F., Kopka J., Jagadish S.V.K., Zuther E. (2021). Physiological and molecular attributes contribute to high night temperature tolerance in cereals. Plant Cell Environ..

[24] Großkinsky D.K., Syaifullah S.J., Roitsch T. (2017). Integration of multi-omics techniques and physiological phenotyping within a holistic phenomics approach to study senescence in model and crop plants. J. Exp. Bot..

[25] Miya M., Yoshikawa T., Sato Y., Itoh J.I. (2021). Genome-wide analysis of spatiotemporal expression patterns during rice leaf development. BMC Genom..

[26] Khan M.M., Jan A., Karibe H., Komatsu S. (2005). Identification of phosphoproteins regulated by gibberellin in rice leaf sheath. Plant Mol. Biol..

[27] Shen F., Wu X., Shi L., Zhang H., Chen Y., Qi X., Wang Z., Li X. (2018). Transcriptomic and metabolic flux analyses reveal shift of metabolic patterns during rice grain development. BMC Syst. Biol..

[28] Molla K.A., Karmakar S., Molla J., Bajaj P., Varshney R.K., Datta S.K., Datta K. (2020). Understanding sheath blight resistance in rice: The road behind and the road ahead. Plant Biotechnol. J..

[29] Ikeda S., Tokida T., Nakamura H., Sakai H., Usui Y., Okubo T., Tago K., Hayashi K., Sekiyama Y., Ono H. (2015). Characterization of leaf blade- and leaf sheath-associated bacterial communities and assessment of their responses to environmental changes in CO_2_, temperature, and nitrogen levels under field conditions. Microbes Environ..

[30] Hirose T., Endler A., Ohsugi R. (1999). Gene expression of enzymes for starch and sucrose metabolism and transport in leaf sheaths of rice (*Oryza sativa* L.) during the heading period in relation to the sink to source transition. Plant Prod. Sci..

[31] Perez C.M., Palmiano E.P., Baun L.C., Juliano B.O. (1971). Starch metabolism in the leaf sheaths and culm of rice. Plant Physiol..

[32] Sengupta S., Mukherjee S., Basak P., Majumder A.L. (2015). Significance of galactinol and raffinose family oligosaccharide synthesis in plants. Front. Plant Sci..

[33] Kavanová M., Lattanzi F.A., Grimoldi A.A., Schnyder H. (2006). Phosphorus deficiency decreases cell division and elongation in grass leaves. Plant Physiol..

[34] Schaarschmidt S., Lawas L.M.F., Glaubitz U., Li X., Erban A., Kopka J., Jagadish S.V.K., Hincha D.K., Zuther E. (2020). Season affects yield and metabolic profiles of rice (*Oryza sativa*) under high night temperature stress in the field. Int. J. Mol. Sci..

[35] Rabbani M.A., Maruyama K., Abe H., Khan M.A., Katsura K., Ito Y., Yoshiwara K., Seki M., Shinozaki K., Yamaguchi-Shinozaki K. (2003). Monitoring expression profiles of rice genes under cold, drought, and high-salinity stresses and abscisic acid application using cDNA microarray and RNA gel-blot analyses. Plant Physiol..

[36] Mukherjee S., Sengupta S., Mukherjee A., Basak P., Majumder A.L. (2019). Abiotic stress regulates expression of galactinol synthase genes post-transcriptionally through intron retention in rice. Planta.

[37] Selvaraj M.G., Ishizaki T., Valencia M., Ogawa S., Dedicova B., Ogata T., Yoshiwara K., Maruyama K., Kusano M., Saito K. (2017). Overexpression of an *Arabidopsis thaliana* galactinol synthase gene improves drought tolerance in transgenic rice and increased grain yield in the field. Plant Biotechnol. J..

[38] Sadok W., Jagadish S.V.K. (2020). The hidden costs of nighttime warming on yields. Trends Plant Sci..

[39] Xie Y., Ravet K., Pearce S. (2021). Extensive structural variation in the Bowman-Birk inhibitor family in common wheat (*Triticum aestivum* L.). BMC Genom..

[40] Othman T., Bakar N.T.A., Abidin R.Z., Mahmood M., Saidi N., Shaharuddin N.A. (2014). Potential of plant’s Bowman-Birk protease inhibitor in combating abiotic stresses: A Mini Review. Bioremediat. Sci. Technol. Res..

[41] Malefo M.B., Mathibela E.O., Crampton B.G., Makgopa M.E. (2020). Investigating the role of Bowman-Birk serine protease inhibitor in Arabidopsis plants under drought stress. Plant Physiol. Biochem..

[42] Seo J.-S., Joo J., Kim M.-J., Kim Y.-K., Nahm B.H., Song S.I., Cheong J.-J., Lee J.S., Kim J.-K., Choi Y.D. (2011). OsbHLH148, a basic helix-loop-helix protein, interacts with OsJAZ proteins in a jasmonate signaling pathway leading to drought tolerance in rice. Plant J..

[43] Park S., Choi M.J., Lee J.Y., Kim J.K., Ha S.-H., Lim S.-H. (2016). Molecular and biochemical analysis of two rice flavonoid 3’-hydroxylase to evaluate their roles in flavonoid biosynthesis in rice grain. Int. J. Mol. Sci..

[44] Marowa P., Ding A., Kong Y. (2016). Expansins: Roles in plant growth and potential applications in crop improvement. Plant Cell Rep..

[45] Le Gall H., Philippe F., Domon J.-M., Gillet F., Pelloux J., Rayon C. (2015). Cell wall metabolism in response to abiotic stress. Plants.

[46] Jones L., McQueen-Mason S. (2004). A role for expansins in dehydration and rehydration of the resurrection plant *Craterostigma plantagineum*. FEBS Lett..

[47] Li F., Han Y., Feng Y., Xing S., Zhao M., Chen Y., Wang W. (2013). Expression of wheat expansin driven by the RD29 promoter in tobacco confers water-stress tolerance without impacting growth and development. J. Biotechnol..

[48] Xu J., Belanger F., Huang B. (2008). Differential gene expression in shoots and roots under heat stress for a geothermal and non-thermal Agrostis grass species contrasting in heat tolerance. Environ. Exp. Bot..

[49] Xu Q., Xu X., Shi Y., Xu J., Huang B. (2014). Transgenic tobacco plants overexpressing a grass *PpEXP1* gene exhibit enhanced tolerance to heat stress. PLoS ONE.

[50] Jadamba C., Kang K., Paek N.-C., Lee S.I., Yoo S.-C. (2020). Overexpression of rice expansin7 (*Osexpa7*) confers enhanced tolerance to salt stress in rice. Int. J. Mol. Sci..

[51] Zhao M.R., Li F., Fang Y., Gao Q., Wang W. (2011). Expansin-regulated cell elongation is involved in the drought tolerance in wheat. Protoplasma.

[52] Han Y., Chen Y., Yin S., Zhang M., Wang W. (2015). Over-expression of *TaEXPB23*, a wheat expansin gene, improves oxidative stress tolerance in transgenic tobacco plants. J. Plant Physiol..

[53] Lü P., Kang M., Jiang X., Dai F., Gao J., Zhang C. (2013). *RhEXPA4*, a rose expansin gene, modulates leaf growth and confers drought and salt tolerance to Arabidopsis. Planta.

[54] Han Z., Liu Y., Deng X., Liu D., Liu Y., Hu Y., Yan Y. (2019). Genome-wide identification and expression analysis of expansin gene family in common wheat (*Triticum aestivum* L.). BMC Genom..

[55] Siahpoosh M.R., Sanchez D.H., Schlereth A., Scofield G.N., Furbank R.T., van Dongen J.T., Kopka J. (2011). Modification of *OsSUT1* gene expression modulates the salt response of rice *Oryza sativa* cv. Taipei 309. Plant Sci..

[56] Do P.T., Degenkolbe T., Erban A., Heyer A.G., Kopka J., Köhl K.I., Hincha D.K., Zuther E. (2013). Dissecting rice polyamine metabolism under controlled long-term drought stress. PLoS ONE.

[57] Haug K., Cochrane K., Nainala V.C., Williams M., Chang J., Jayaseelan K.V., O’Donovan C. (2019). MetaboLights: A resource evolving in response to the needs of its scientific community. Nucleic Acids Res..

[58] Chomczynski P., Sacchi N. (2006). The single-step method of RNA isolation by acid guanidinium thiocyanate-phenol-chloroform extraction: Twenty-something years on. Nat. Protoc..

[59] Zuther E., Schulz E., Childs L.H., Hincha D.K. (2012). Clinal variation in the non-acclimated and cold-acclimated freezing tolerance of *Arabidopsis thaliana* accessions. Plant Cell Environ..

[60] Barrett T., Wilhite S.E., Ledoux P., Evangelista C., Kim I.F., Tomashevsky M., Marshall K.A., Phillippy K.H., Sherman P.M., Holko M. (2013). NCBI GEO: Archive for functional genomics data sets-update. Nucleic Acids Res..

[61] Andrews S., FASTQC (2010). A Quality Control Tool for High Throughput Sequence Data [Online]. http://www.bioinformatics.babraham.ac.uk/projects/fastqc/.

[62] Bolger A.M., Lohse M., Usadel B. (2014). Trimmomatic: A flexible trimmer for Illumina sequence data. Bioinformatics.

[63] Dobin A., Davis C.A., Schlesinger F., Drenkow J., Zaleski C., Jha S., Batut P., Chaisson M., Gingeras T.R. (2012). STAR: Ultrafast universal RNA-seq aligner. Bioinformatics.

[64] Love M.I., Huber W., Anders S. (2014). Moderated estimation of fold change and dispersion for RNA-seq data with DESeq2. Genome Biol..

[65] Robinson M.D., McCarthy D.J., Smyth G.K. (2010). edgeR: A Bioconductor package for differential expression analysis of digital gene expression data. Bioinformatics.

[66] Benjamini Y., Hochberg Y. (1995). Controlling the false discovery rate: A practical and powerful approach to multiple testing. J. R. Stat. Soc. Ser. B.

[67] R Core Team (2018). R: A Language and Environment for Statistical Computing, Version 3.4.2.

[68] R StudioTeam (2020). RStudio: Integrated Development for R.

[69] Stacklies W., Redestig H., Scholz M., Walther D., Selbig J. (2007). pcaMethods—A Bioconductor package providing PCA methods for incomplete data. Bioinformatics.

[70] Wickham H. (2016). ggplot2: Elegant Graphics for Data Analysis.

