# Supplementary material for: Differentiation of the High Night Temperature Response in Leaf Segments of Rice Cultivars with Contrasting Tolerance
Source: Int J Mol Sci. 2021 Sep 28;22(19):10451. doi: 10.3390/ijms221910451 (PMC8508630; doi:10.3390/ijms221910451)
Supplement: Supplementary file 1 [file ijms-22-10451-s001.zip › Schaarschmidt and Glaubitz 2021 Suppl_Fig.pdf]

Schaarschmidt and Glaubitz et al. 2021, Suppl. Figures

Suppl. Fig. S1

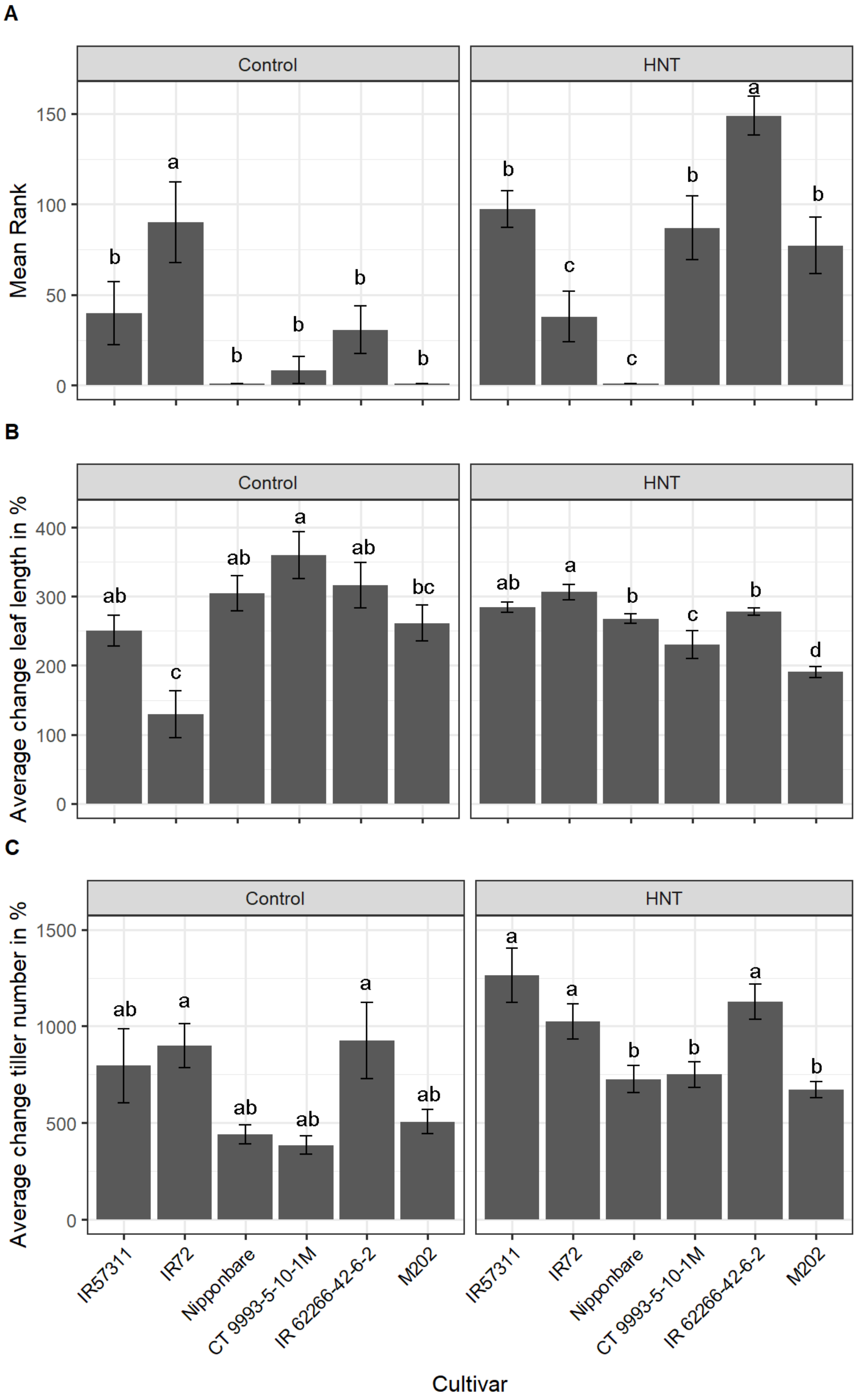

Figure S1. Mean rank of necrosis scores (A), average change of leaf length (B) and average change of tiller number (C) of six *Oryza sativa* cultivars at control and HNT conditions. Cultivars are sorted based on chlorosis ranking under HNT. For the necrosis rank larger values indicate high damage while smaller values represent a lower damage. Necrosis mean ranks are based on visual scoring in percentage.

Suppl. Fig. S2

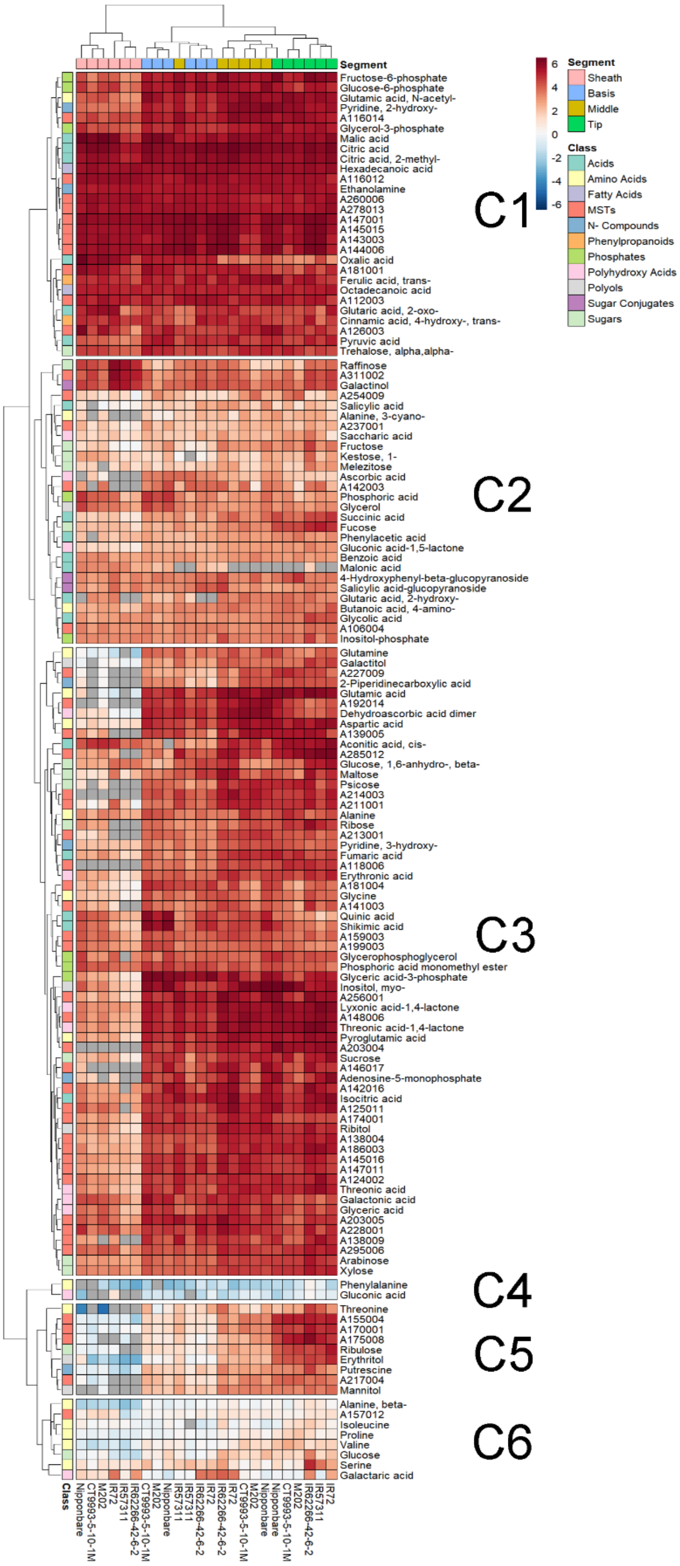

Figure S2. Metabolites in leaf sheath and three leaf blade segments represented as log_2 transformed levels. Red color indicates a higher level, blue color a lower level. Tolerance and metabolite classes are color-coded.

Suppl. Fig. S3

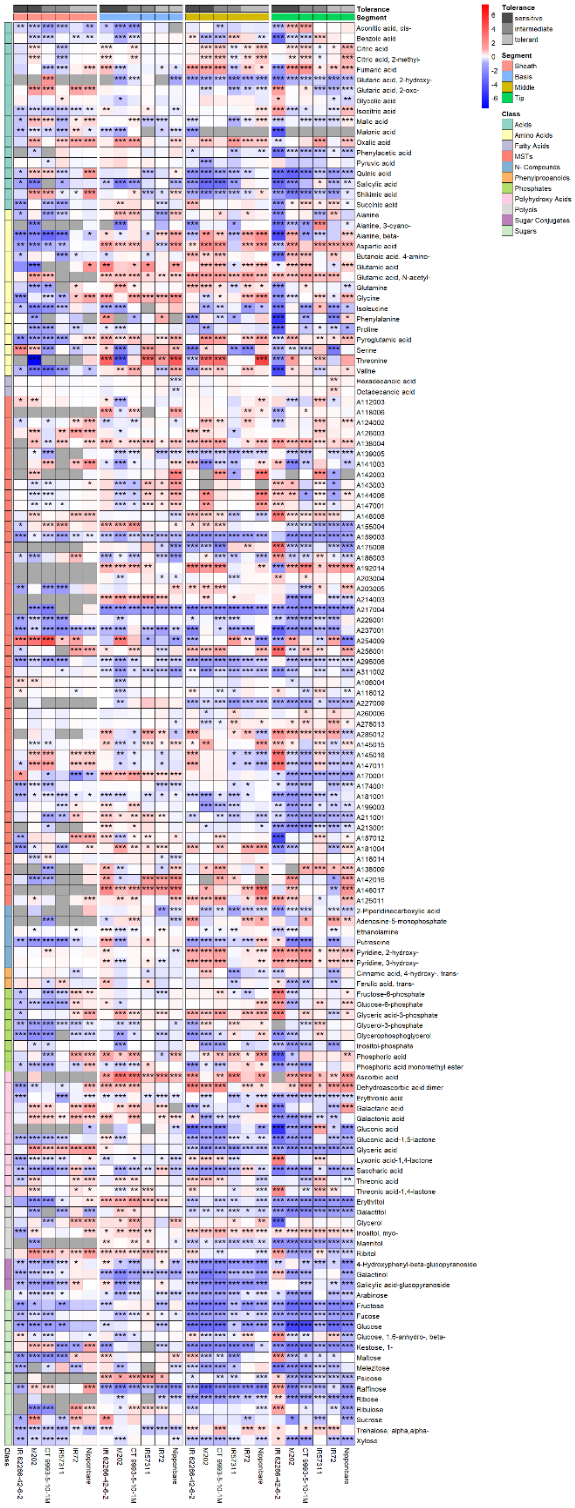

Figure S3. Changes of metabolite levels in leaf sheath and three leaf blade segments represented as log_2 fold changes. Red color indicates a higher log_2 fold change, blue color a lower log2 fold change compared to control. Asterisks display significance level of p values: < 0.001 - ***, >= 0.001 and <= 0.01 - **, >= 0.01 and <= 0.05 - *. Tolerance and metabolite classes are color coded.
